# Using Corneal Confocal Microscopy to Identify Therapeutic Agents for Diabetic Neuropathy

**DOI:** 10.3390/jcm11092307

**Published:** 2022-04-21

**Authors:** Corinne G. Jolivalt, May Madi Han, Annee Nguyen, Fiona Desmond, Carlos Henrique Alves Jesus, Daniela C. Vasconselos, Andrea Pedneault, Natalie Sandlin, Sage Dunne-Cerami, Katie E. Frizzi, Nigel A. Calcutt

**Affiliations:** Department of Pathology, University of California San Diego, La Jolla, CA 92093, USA; cjolivalt@ucsd.edu (C.G.J.); mmh015@health.ucsd.edu (M.M.H.); annee.nguyen.1996@gmail.com (A.N.); fionajdesmond@gmail.com (F.D.); carlos.alves.jesus@hotmail.com (C.H.A.J.); daniscalcovasconcelos@gmail.com (D.C.V.); apedneau@ucsd.edu (A.P.); nsandlin@udallas.edu (N.S.); sagedunnecerami@gmail.com (S.D.-C.); kfrizzi@health.ucsd.edu (K.E.F.)

**Keywords:** diabetic neuropathy, corneal confocal microscopy, CNTF, GLP-1, exendin-4, muscarinic antagonist, cyclopentolate, glycopyrrolate, gallamine

## Abstract

Corneal confocal microscopy (CCM) is emerging as a tool for identifying small fiber neuropathy in both peripheral neuropathies and neurodegenerative disease of the central nervous system (CNS). The value of corneal nerves as biomarkers for efficacy of clinical interventions against small fiber neuropathy and neurodegenerative disease is less clear but may be supported by preclinical studies of investigational agents. We, therefore, used diverse investigational agents to assess concordance of efficacy against corneal nerve loss and peripheral neuropathy in a mouse model of diabetes. Ocular delivery of the peptides ciliary neurotrophic factor (CNTF) or the glucagon-like peptide (GLP) analog exendin-4, both of which prevent diabetic neuropathy when given systemically, restored corneal nerve density within 2 weeks. Similarly, ocular delivery of the muscarinic receptor antagonist cyclopentolate protected corneal nerve density while concurrently reversing indices of systemic peripheral neuropathy. Conversely, systemic delivery of the muscarinic antagonist glycopyrrolate, but not gallamine, prevented multiple indices of systemic peripheral neuropathy and concurrently protected against corneal nerve loss. These data highlight the potential for use of corneal nerve quantification by confocal microscopy as a bridging assay between in vitro and whole animal assays in drug development programs for neuroprotectants and support its use as a biomarker of efficacy against peripheral neuropathy.

## 1. Introduction

Corneal confocal microscopy (CCM), originally developed for use in ophthalmology, is emerging as an important, non-invasive tool for identifying small fiber neuropathy in diverse clinical conditions. The capacity to visualize corneal nerves of the sub basal plexus and stroma in real time and without requiring use of contrast agents allows for longitudinal studies that can track nerve density and arborization in the same subjects over time [1]. The nerves visualized are comprised of peripheral C and Aδ sensory neurons (reviewed in [2]) with cell bodies located in the trigeminal ganglion and may be considered analogous to the sensory neurons that innervate the skin epidermis, albeit with shorter axons and experiencing a somewhat different tissue environment at their peripheral terminals. The vulnerability of corneal neurons to systemic metabolic and toxic insults appears similar to that of other sensory neurons, making them a potential biomarker for sensory peripheral neuropathy that avoids the invasive surgeries required to collect nerve or skin biopsies. Loss of corneal nerves has been reported in a variety of peripheral neuropathies with small fiber involvement, including those associated with chemotherapy [3], HIV [4], Fabry’s disease [5], and Charcot-Marie-Tooth disease [6], along with idiopathic small fiber neuropathy [7]. The most extensive characterization to date has been in diabetic neuropathy where initial reports of corneal nerve loss [8,9] have been amplified and extended to convincingly document its utility as a biomarker that is reproducible and sensitive enough to identify sub-clinical neuropathy and track progression of neuropathy (reviewed in [10]). There have also been reports of corneal nerves depletion in subjects with neurodegenerative disease of the central nervous system such as Parkinson’s disease [11], mild cognitive impairment [12], frontotemporal dementia [13], and amyotrophic lateral sclerosis [14].

The extent to which corneal nerves can be used to track efficacy of interventions is less well documented. Morphometric parameters of corneal nerve injury, such as reduced nerve density and length, have been reported to improve in diabetic subjects following institution of enhanced metabolic control via intensive insulin therapy [15] or combined pancreas and kidney transplantation, with the latter resulting in improvements to corneal nerve parameters that preceded detectable improvement in established measures of neuropathy [16]. The absence of therapies that directly target diabetic neuropathy has impeded evaluation of the viability of corneal nerves as a biomarker for drug intervention, although there is limited evidence from exploratory clinical trials. For example, type 2 diabetic subjects treated with an analog of erythropoietin showed improvement in neuropathic symptoms that was accompanied by increased corneal nerve fiber density in a subset of subjects with the most extreme baseline corneal nerve loss [17]. Corneal nerve fiber length was also improved in type 1 diabetic subjects treated with omega-3 polyunsaturated fatty acids for 12 months [18]. However, it should be noted that there was no concurrent effect of either treatment on objective measures of diabetic neuropathy, such as nerve conduction slowing or sensory dysfunction, so that it is not clear if corneal nerves were serving as a sensitive biomarker whose repair preceded other measures or were responding to the therapy independent of effects on systemic neuropathy.

Imaging of corneal nerves in other species using confocal microscopy is technically viable [19], although the smaller diameter of the visualized nerves in rodents commonly used in laboratory research makes automated quantification challenging [20]. Rat and mouse models of type 1 or type 2 diabetes develop reduced density and/or length of corneal nerves in the sub-basal plexus within weeks-months of onset of hyperglycemia [21,22,23]. The precise time to onset of loss appears to vary with severity of insulinopenia and other metabolic factors and may be preceded by a transient elevation in density of the sub-basal nerve plexus [21,24,25]. The presence of a corneal nerve lesion in commonly studied rodent models of diabetes that also develop other features of clinical neuropathy, such as sensory loss and nerve conduction slowing, offers an opportunity to explore the use of corneal nerve integrity as an efficacy biomarker following systemic delivery of exploratory therapeutic interventions. It also suggests use as an in vivo efficacy screening assay that bridges in vitro screening studies and in vivo systemic efficacy studies of a drug development funnel [26]. We, therefore, assessed the efficacy of potential therapeutic agents, delivered topically to the eye or systemically, to test the biomarker potential of corneal nerves in diabetic neuropathy.

## 2. Materials and Methods

### 2.1. Animals

All studies were performed using adult female Swiss Webster mice (Envigo, Livermore, CA, USA) using protocols approved by the IACUC of the University of California San Diego (S02059M). Mice were maintained 4–5/cage on Tek-fresh bedding (Envigo, Livermore, CA, USA) in a AAALAC-approved vivarium under a 12 h light:dark cycle with free access to water and food (Teklad 5001 diet, Envigo, Livermore, CA, USA). Insulin-deficient diabetes was induced by injection of streptozotocin (100 mg/kg i.p. freshly dissolved in 0.9% sterile saline) on two consecutive days, with each injection following an overnight fast [20]. Hyperglycemia was confirmed 4–5 days later, and at various points during the duration of each study, by measuring glucose levels in a drop of blood obtained by tail prick using a strip operated meter. Only mice with blood glucose levels that remained at or above 15 mmol/L for the duration of the study were included as diabetic. Drugs used for treatment of neuropathy were ciliary neurotrophic factor (CNTF: in 0.9% sterile saline, Regeneron), the glucacon-like peptide1 (GLP-1) analog exendin-4 (in 0.9% sterile saline; American Peptide Company, Sunnyvale, CA, USA ), cyclopentolate (in phosphate buffered saline, Sigma-Aldrich), glycopyrrolate (in 0.9% sterile saline, Selleck Chemicals Inc.), and gallamine (in 0.9% sterile saline, Sigma-Aldrich). Topical treatment with either CNTF (25 ng/mL in 0.9% sterile saline), the GLP-1 agonist exendin-4 (100 ng/mL in 0.9% sterile saline), or vehicle was applied daily (Monday–Friday) as a single 50 µL drop to one eye for the last 2 weeks of diabetes. Cyclopentolate (50 µL of 0.5%, 1.0% or 2.0% in phosphate buffered saline) or vehicle were delivered daily (Monday-Friday) as a single 50 µL drop to one eye for the final 4 weeks of diabetes. Glycopyrrolate (10 mg/kg/day, s.c. in 0.9% sterile saline), gallamine (1.0 mg/kg/day in 0.9% sterile saline), or vehicle were delivered daily (Monday–Friday) to diabetic mice by sub-cutaneous injection for 12 weeks, starting at onset of diabetes.

### 2.2. Corneal Confocal Microscopy

The procedures for adapting a clinical corneal confocal microscope for use in rodents is described in technical detail elsewhere [20]. Briefly, mice were anesthetized with 2.5–4.0 ppm isoflurane in an induction chamber then placed on a custom-built small animal platform, where general anesthesia was maintained via a face mask. A strap was used to secure the mouse with its head tilted so that the eye faced the objective of the corneal confocal microscope (HRT3 with Rostock corneal module; Heidelberg Engineering, Heidelberg, Germany). GenTeal eye gel (Alcon Inc., Fort Worth, TX, USA) was applied to prevent the eyes from drying and the tomocap of the microscope positioned to make contact with the gel without depressing the cornea. A volume scan of 40 consecutive images at 2 µm depth intervals was collected between the corneal epidermis and the stroma and stored for analysis. Of these, 5 consecutive images (each being 384 × 384 pixels with 1 µm lateral resolution) of the sub basal nerve plexus and 10 consecutive images of the superficial stroma were subsequently analyzed for area of nerve present using an electronic tracing pen and ImageJ software. All nerves visualized were traced by a trained observer unaware of the animal or treatment group and quantified either as % occupancy (yes/no for presence of nerve) using an 8 × 8 grid placed on each image [20,21] or as total nerve length, in pixels of nerve traced, per image [27]. Prior studies have shown that these two methods give similar findings in diabetic mice [27].

### 2.3. Motor Nerve Conduction Velocity

The protocol is described in detail elsewhere [20]. Briefly, mice we anesthetized with 2.5–4.0 ppm isoflurane and core temperature maintained at 37 °C. The sciatic nerve was stimulated (0.05 ms, 200 mV; Powerlab 4/30 stimulator; AD Instruments) via needle electrodes placed at the sciatic notch and Achilles tendon with resulting electromyograms recorded on a computer running oscilloscope emulation software (LabChart Pro; AD Instruments, Colorado Springs, CO, USA) via needle electrodes placed in the ipsilateral interosseus muscles. Motor nerve conduction velocity (MNCV) was calculated using the peak–peak latency between pairs of M waves and the distance between the two stimulation sites. All measurements were made in triplicate, with the median used to represent values for that animal.

### 2.4. Paw Heat Sensation

The protocol is described in detail elsewhere [20]. Briefly, mice were placed in plexiglass cubicles on top of a Hargreaves testing device (UARD, La Jolla, CA, USA) calibrated to deliver a heating rate of 1 °C/s, which selectively activates C fibers [28]. Mice were allowed to acclimate for 15 min on a glass surface maintained at 30 °C. A focused heat source was then placed directly below the center of one hind paw, activated, and the time until paw withdrawal recorded. The response latency was measured three times at 5-min intervals and the median value used to represent the response latency.

### 2.5. Paw Tactile Sensation

The protocol is described in detail elsewhere [20]. Mice were placed within an observation chamber with a wire mesh floor and allowed to acclimate for 15 min. Von Frey filaments were applied in ascending sequence (3.22, 3.61, 3.84, 4.08, 4.31, 4.56, and 4.74) until the first withdrawal response was evoked, after which the up-down method was applied [29]. The sequence of negative and positive responses was used to calculate 50% paw withdrawal threshold (50% PWT). Paws were tested in triplicate with the median used to represent the 50% PWT for that animal.

### 2.6. Statistical Analysis

All studies were performed on coded animals, tissue, and images. Between group comparisons were made by one way ANOVA with Dunnett’s post hoc test using Prism statistical software (GraphPad Software LLC, San Diego, CA, USA). Data are presented as group mean ± SEM.

## 3. Results

### 3.1. General Physiology

The systemic impact of diabetes and treatments is shown in Table 1. STZ injection induced marked hyperglycemia that was not impacted by any treatment. Diabetic mice showed only a mild restriction of weight gain and no exogenous insulin was administered to any mice at any point. Body weight of diabetic mice was not impacted by treatment other than 12 weeks of systemic gallamine, which prevented any weight gain so that values were significantly (*p* < 0.05) lower than vehicle-treated diabetic mice at study end.

### 3.2. Topical Delivery of Peptides

**Figure 1:**
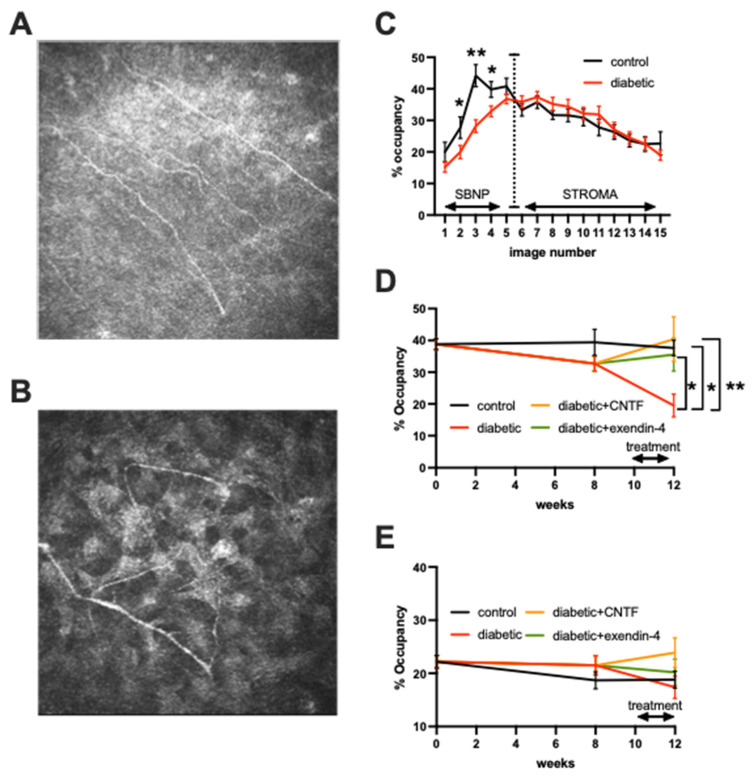
Reversal of corneal neuropathy by topical CNTF or exendin-4 in diabetic mice. Representative images of sub-basal nerve plexus (**A**) and stromal nerves (**B**) from Swiss Webster mice. Quantification of nerve % occupancy (**C**) of 15 consecutive individual images collected at 2 µm intervals from the corneal epithelium to the stroma in control mice and mice with 4 weeks of diabetes. Data points are group mean ± SEM of N = 9 control and N = 23 diabetic mice. * = *p* < 0.05 and ** = *p* < 0.01 by unpaired test. Quantification of sub-basal nerve plexus (**D**) and stromal nerve (**E**) % occupancy over time in control (N = 9) mice, diabetic mice (N = 9), and diabetic mice treated daily for the last 2 weeks with eyedrops containing CNTF (N = 7) or exendin-4 (N = 7). Data points are group mean ± SEM with between group analysis at study end by one way ANOVA with Dunnett’s post hoc test. * = *p* < 0.05 and ** = *p* < 0.01 vs. vehicle treated diabetic mice. Data points are group mean ± SEM with between group analysis by one way ANOVA followed by Dunnett’s Post hoc test. * = *p* < 0.05 and ** = *p* < 0.01 vs. vehicle treated diabetic mice.

Quantification of 15 consecutive images of the cornea, spanning the sub-basal nerve plexus (Figure 1A) and superficial stroma (Figure 1B) indicated significantly reduced nerve occupancy after 4 weeks of diabetes that was limited to the middle of the sub-basal nerve plexus (Figure 1C). Additional collection of images at week 8 of diabetes was followed by topical treatment with either CNTF, the GLP-1 agonist exendin-4, or vehicle alone for the last 2 weeks of the 12-week study period (Table 2). At study end, vehicle-treated diabetic mice had a significantly (*p* < 0.05) reduced density of nerves in the sub-basal plexus compared to vehicle-treated control mice and diabetic mice treated with either CNTF or exendin-4 (Figure 1D). Nerve density in the superficial stroma was not significantly different between any group at study end (Figure 1E).

### 3.3. Topical Delivery of a Muscarinic Antagonist

**Figure 2:**
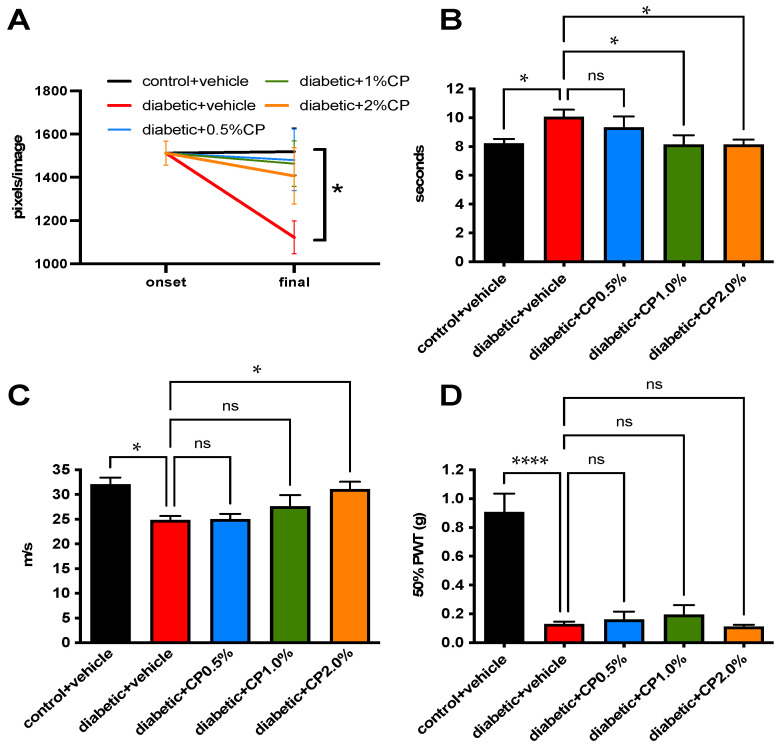
Prevention of neuropathy by topical cyclopentolate in diabetic mice. Sub-basal nerve plexus density (**A**), paw response latency to heat (**B**), sciatic motor nerve conduction velocity (**C**), and paw 50% response threshold to von Frey filaments (**D**) after 8 weeks of diabetes, with daily treatment to the eye with vehicle or cyclopentolate (CP) for the last 4 weeks. Data points are group mean ± SEM with between group analysis by one way ANOVA followed by Dunnett’s Post hoc test. * = *p* < 0.05 and **** = *p* < 0.0001 vs. vehicle-treated diabetic mice. ns: not significant.

Eight weeks of diabetes caused a marked decline in nerve density of the sub-basal plexus that was prevented by topical treatment with cyclopentolate to one eye (Table 2) so that only diabetic mice treated with vehicle had values significantly (*p* < 0.05) different from vehicle-treated control mice at study end (Figure 2A). Cyclopentolate also caused dose-dependent alleviation of small fiber mediated paw heat hypoalgesia (Figure 2B) and large fiber MNCV slowing (Figure 2C), but did not prevent large-fiber mediated allodynia measured with von Frey filaments (Figure 2D).

### 3.4. Systemic Delivery of a Muscarinic Antagonist

**Figure 3:**
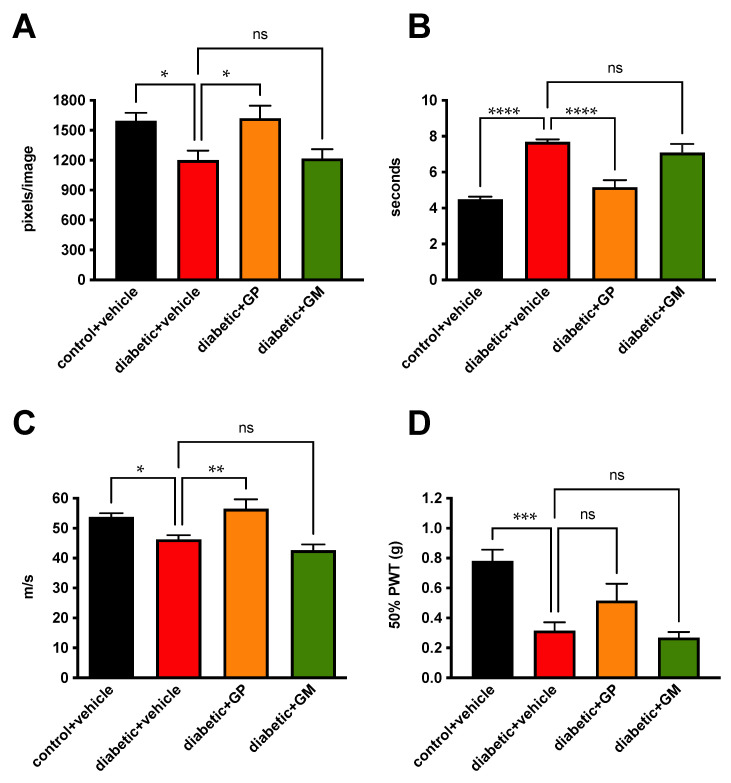
Prevention of neuropathy by systemic glycopyrrolate in diabetic mice. Sub-basal nerve plexus density (**A**), paw response latency to heat (**B**), sciatic motor nerve conduction velocity (**C**), and paw 50% response threshold to von Frey filaments (**D**) after 12 weeks of diabetes, with daily treatment to the eye with vehicle, glycopyrrolate (GP), or gallamine (GM). Data points are group mean ± SEM with between group analysis by one way ANOVA followed by Dunnett’s post hoc test. * = *p* < 0.05, ** = *p* < 0.01, *** = *p* < 0.001 and **** = *p* < 0.0001 vs. vehicle-treated diabetic mice. ns: not significant.

Diabetic mice were treated with glycopyrrolate, gallamine, or vehicle for 12 weeks, starting at onset of diabetes (Table 2). Reduced nerve density in the sub-basal plexus of vehicle-treated diabetic mice (*p* < 0.05) was prevented by glycopyrrolate but not gallamine (Figure 3A). This was accompanied by prevention of small fiber mediated paw heat hypoalgesia (Figure 3B) and large fiber MNCV slowing (Figure 3C) by glycopyrrolate but not gallamine, whereas large fiber mediated allodynic responses to von Frey filaments were not different from vehicle treated diabetic mice after treatment with either agent (Figure 3D).

## 4. Discussion

Diabetic rats and mice are widely used in development of potential therapeutics against diabetic neuropathy because they display functional disorders, such as nerve conduction slowing and loss of heat sensation, that also represent diagnostic features of the human condition (reviewed in [26]). In contrast, structural damage at the sciatic nerve level is limited, takes many months to develop, and lacks overt hallmarks of human diabetic neuropathy, such as segmental demyelination and Wallerian degeneration. A reduction in the number of small sensory neuron terminal regions in skin epidermis provides a structural correlate to human small fiber diabetic neuropathy. However, as in humans, tissue collection for standard histological imaging is invasive and not amenable to longitudinal studies in the same animal. Loss of nerves in the corneal sub-basal plexus of diabetic rodents and humans may have value for assessing neuroprotective and neuroregenerative properties of potential therapeutics, provided that efficacy in corneal sub-basal nerves is predictive of efficacy against other indices of systemic neuropathy.

Topical delivery of the neurotrophic factor CNTF and the GLP-1 analog exendin-4 to the eye of diabetic mice was used to explore use of corneal confocal microscopy as a bridge between in vitro studies that assess the capacity of agents to promote neurite outgrowth in sensory neuron cultures and studies that use systemic treatment to prevent or reverse indices of peripheral neuropathy in diabetic rodents. We have previously shown that topical delivery of insulin or IGF-1 to the eye prevented or reversed corneal nerve loss in diabetic mice without impacting systemic blood glucose levels, indicating that corneal nerves can illustrate neurotrophic properties of locally delivered peptides [21,30]. Like insulin and IGF-1 [30,31], both CNTF [32,33] and GLP-1 analogs [34,35] exhibit neurotrophic/neuroprotective properties in adult primary sensory neurons in vitro. They also protect against sensory loss and nerve conduction slowing when delivered systemically to diabetic rodents [34,35,36,37,38]. We are not aware of prior reports of either CNTF or exendin-4 impacting corneal nerve density in diabetic rodents, although CNTF delivered topically to the eye restored rates of corneal wound healing and re-innervation in diabetic rodents following corneal lesions [39,40]. In the present study, iterative corneal confocal microscopy allowed tracking of corneal nerve density from before the onset of diabetes to a point where deficits were present in diabetic mice and the response to subsequent interventional treatment. It was notable from volume stack data that reduced corneal nerve density was limited to the sub-basal nerve plexus and that stromal nerve density was unaffected by diabetes at any point, indicative of a dying back neuropathy. Both CNTF and exendin-4, when applied by eye drop once daily, 5 days a week for 2 weeks, completely restored corneal nerve density. The short treatment period and small amounts of peptide required to demonstrate efficacy may encourage use of this assay to bridge in vitro observations and in vivo systemic efficacy assays, particularly where supplies of an experimental agent may be limited and not immediately amenable to whole animal systemic delivery studies.

We have previously reported that muscarinic antagonists that selectively or specifically target the M_1_ receptor sub-type (M_1_R) promote neurite growth in adult sensory neurons in vitro [41,42,43,44] and correct multiple indices of neuropathy when given systemically to diabetic rodents [41,45]. Moreover, topical delivery of the M_1_R-specific antagonist MT7 or the non-specific muscarinic antagonist atropine to the eye of diabetic mice reversed or prevented reduced corneal nerve density in diabetic rodents [44,45]. In the present study, we extended our investigations of topical delivery of muscarinic antagonists to cyclopentolate, a non-selective muscarinic antagonist that is widely used as a relatively short-acting pupil dilator in clinical ophthalmic procedures. Prevention of corneal nerve loss by 0.5–2.0% solutions of cyclopentolate was accompanied by dose-dependent prevention of MNCV slowing and paw heat hypoalgesia without impact on the severity of diabetes. This suggests that there was systemic distribution of drug applied to the eye and re-enforces associations between therapeutic effects on corneal nerves and on indices of system-wide sensory and motor neuropathy. There is precedence for peptides applied to the eye having systemic actions [46]. Speculation that the widespread clinical use of cyclopentolate may promote unnecessary nerve growth in the cornea requires an appreciation of whether it enhances nerve growth in the cornea of non-diabetic rodents and the minimum number of treatments required to initiate an effect. At present, we have not performed such studies, although they are clearly viable using iterative corneal confocal microscopy.

We next determined whether systemic delivery of muscarinic antagonists to diabetic mice could impact corneal nerve density, as this represents the most likely scenario for use of corneal nerves as a biomarker of the efficacy of therapeutic agents in clinical trials. Prior studies using systemic delivery of the M_1_R-selective antagonist pirenzepine have shown efficacy against multiple indices of diabetic peripheral neuropathy in rodents [41,45], but did not evaluate corneal nerves. We elected to use glycopyrrolate as it is widely used in clinical practice as a non-selective muscarinic receptor antagonist that, like pirenzepine, does not cross the blood–brain barrier to disrupt CNS function. Efficacy in preventing MNCV slowing and paw heat hypoalgesia was accompanied by protection of sub-basal nerve plexus density in the absence of impact on the severity of diabetes. In contrast, the M_2_R-selective antagonist gallamine was without effect on any measure of peripheral neuropathy, which is consistent with its lack of impact on neurite outgrowth from sensory neurons in vitro [41] and the focus of neuroprotective properties to molecules that antagonize the M_1_R [42,44]. These data further support use of corneal nerves as a biomarker for efficacy of therapeutics on multiple aspects of diabetic neuropathy and joins similarly supportive data in diabetic rodents treated systemically with ilepatril, a vasopeptidase inhibitor [23], or menhaden oil, which is rich in n-3 fatty acids [47,48,49].

Despite ameliorating indices of both small and large fiber diabetic neuropathy, neither 12 weeks of systemic glycopyrrolate nor 4 weeks of ocular cyclopentolate prevented tactile allodynia in diabetic mice. This representation of neuropathic pain was also not prevented by the non-selective muscarinic receptor antagonist atropine when applied to the eye or paw of diabetic mice [45]. In contrast, the selective M_1_R antagonist pirenzepine given either systemically [41] or topically to the paw [45] has been reported to prevent allodynia. Pirenzepine does not have acute analgesic effects in diabetic rodents [41], so this dichotomy may reflect the M_1_R selectivity of pirenzepine compared to the other anti-muscarinic agents in regulating pain phenotype. It should also be noted that a prior study where atropine was applied to the paw of diabetic mice to provide low dose systemic distribution found efficacy against MNCV slowing and paw thermal hypoalgesia in diabetic mice, but not against loss of corneal nerves [45]. This may reflect the limited amount of atropine in circulation and accessing the cornea and/or trigeminal ganglia. Nevertheless, it also serves to highlight a residual concern regarding use of corneal nerves as a biomarker for systemic drug efficacy against diabetic neuropathy-namely that access of drug to the corneal nerve terminals and/or their perikarya must be equivalent to access to other peripheral nerves.

In summary (see Table 2), we have provided novel data showing efficacy of CNTF, exendin-4, and cyclopentolate in preventing or restoring diabetes-induced depletion of the corneal sub-basal nerve plexus when delivered to the eye. Delivery of cyclopentolate to the eye also impacted functional indices of large and small fiber peripheral neuropathy in diabetic mice, while systemic delivery of glycopyrrolate both prevented onset of the same functional indices of peripheral neuropathy and also protected against corneal nerve loss. Although the present studies are limited to animal models of diabetic neuropathy, there is emerging evidence of similar utility of corneal confocal microscopy in rodent models of other peripheral neuropathies. For example, studies in preclinical models of HIV-associated neuropathy have shown that M_1_R antagonists prevent impaired neurite outgrowth In vitro, prevent and reverse loss of corneal nerves when applied topically to the eye, and prevent NCV slowing, paw heat hypoalgesia, and loss of corneal nerves following systemic delivery [41,50]. Similarly, muscarinic receptor antagonists prevented reduced neurite outgrowth in vitro following exposure to the chemotherapeutic agents paclitaxel and oxaliplatin [41] and also prevented loss of corneal sub-basal nerves when delivered to the eye of mice with oxalipatin-induced neuropathy [44]. Whether systemic delivery of muscarinic antagonists also protects both peripheral nerve function and corneal nerve density in mice with chemotherapy-induced peripheral neuropathy is not known, but such studies are encouraged by both the in vitro and in vivo ocular delivery findings. Taken together, data obtained across multiple models of peripheral neuropathy highlight the potential of measuring corneal nerve density by confocal microscopy to serve as a bridging assay for in vivo efficacy of potential therapeutics that are initially identified by in vitro neurite growth screening assays, prior to advancement to systemic delivery studies. They also add support to the use of corneal nerve quantification, not only as a biomarker of diabetic neuropathy, but also of efficacy of therapeutic interventions

## Figures and Tables

**Table 1:** General physiological parameters in all studies.

	N	Body Weight (g)		Blood Glucose (mmol/L)	
		Onset of treatment	Final	Onset of treatment	Final
Control + vehicle	9	30.9 ± 0.9	30.5 ± 1.0 *	8.3 ± 0.7 ***	7.4 ± 0.3 ***
Diabetic + vehicle	9	28.0 ± 1.2	27.1 ± 0.9	31.5 ± 1.1	32.3 ± 0.6
Diabetic + CNTF	7	25.4 ± 1.1 *	25.4 ± 1.1	31.7 ± 0.7	32.5 ± 0.5
Diabetic + exendin-4	7	25.6 ± 0.7 *	26.3 ± 0.8	32.8 ± 0.4	31.9 ± 0.8
Control + vehicle	10	25.0 ± 0.4	24.8 ± 0.3	7.6 ± 0.2 ***	9.2 ± 0.3 ***
Diabetic + vehicle	8	23.3 ± 0.8	24.3 ± 0.6	23.6 ± 1.6	37.8 ± 0.9
Diabetic + 0.5% CP	8	23.7 ± 0.5	23.9 ± 0.4	23.5 ± 1.7	34.4 ± 1.9
Diabetic + 1.0% CP	9	22.4 ± 0.5	24.7 ± 0.4	22.8 ± 1.1	35.1 ± 1.5
Diabetic + 2.0% CP	9	23.2 ± 0.4	23.9 ± 0.8	23.7 ± 1.0	34.8 ± 1.7
Control + vehicle	9	26.8 ± 0.5	30.4 ± 0.6 *	8.0 ± 0.2 ***	8.4 ± 0.4 ***
Diabetic + vehicle	10	26.1 ± 0.6	28.4 ± 0.5	23.7 ± 1.1	35.1 ± 1.3
Diabetic + GP	10	25.7 ± 0.5	27.0 ± 0.8	25.7 ± 1.1	36.4 ± 1.5
Diabetic + GM	10	25.4 ± 0.5	25.3 ± 0.5 *	21.0 ± 1.4	35.5 ± 1.6
Statistical significance		* = *p* < 0.05 vs. diabetic	* = *p* < 0.05 vs. diabetic	*** = *p* < 0.001 vs. diabetic	*** = *p* < 0.001 vs. diabetic

Data are group mean ±SEM with statistical comparisons vs. the diabetic + vehicle group by one way ANOVA with Dunnett’s post-hoc test. * *p* < 0.05 and *** *p* < 0.001 vs. respective diabetic+vehicle group. CP = cyclopentolate, GP = glycopyrrolate, GM = gallamine.

**Table 2:** Summary of treatments and their efficacy against indices of neuropathy in diabetic mice. ND = not determined.

Treatment	Dose	Diabetes Duration	Duration of Treatment	Efficacy vs. Corneal Nerve Loss	Efficacy vs. MNCV Slowing	Efficacy vs. Heat Hypoalgesia	Efficacy vs. Tactile Allodynia
CNTF	50 µL, 25 ng/mL (topical to eye)	10 weeks	Daily, last 2 weeks of diabetes	√	ND	ND	ND
Exendin-4	50 µL, 100 ng/mL (topical to eye)	10 weeks	Daily, last 2 weeks of diabetes	√	ND	ND	ND
Cyclopentolate	50 µL 0.5–2.0% (topical to eye)	8 weeks	Daily, last 4 weeks of diabetes	√	√ (dose dependent)	√ (dose dependent)	X
Glycopyrrolate	10 mg/kg/day (subcutaneous)	12 weeks	Daily, 12 weeks	√	√	√	X
Gallamine	1 mg/kg/day (subcutaneous)	12 weeks	Daily, 12 weeks	X	X	X	X

## Data Availability

The data presented in this study are available on acceptable request from the corresponding author.
